# Can Distributed Ledgers Help to Overcome the Need of Labeled Data for Agricultural Machine Learning Tasks?

**DOI:** 10.34133/plantphenomics.0070

**Published:** 2023-07-10

**Authors:** Stefan Paulus, Benjamin Leiding

**Affiliations:** ^1^ Institute of Sugar Beet Research, Holtenser Landstr. 77, 37079 Göttingen, Germany.; ^2^ Institute for Software and Systems Engineering, TU Clausthal, Wallstr. 6, 38640 Goslar, Germany.

## Introduction

Plant phenotyping describes the result of the interaction of genotype with the environment [1]. This is performed with high throughput in greenhouses by automated screening systems using different types of imaging and non-imaging sensors [2]. The high-throughput imaging routines result in large amounts of data, which require sophisticated processing routines. Sharing and reusing phenotype-related data are not common, because its acquisition and processing are resource costly and technically intensive [3].

General standards for data acquisition and even specific phenotyping-related standards exist following the approach for Minimum Information About a Plant Phenotyping Experiment (MIAPPE) [4]. Moreover, recently, funding organizations have integrated the FAIR (findability, accessibility, interoperability, and reusability) principles for data management and made it mandatory [5].

Today, acquiring data is always followed by machine learning (ML), and supervised learning in particular depends on adequately preprocessed data such as data labeling. Especially with the establishment of deep learning routines, the need for large quantities of high quality, labeled, data has increased and subsequently caused a bottleneck for model training in plant phenotyping [6]. Similar to the underlying datasets, standardization, availability, and quality requirements also pose a challenge to the trained models.

Open datasets showed their usability, e.g., in computer vision and ML challenges, like leaf segmentation and counting [7]. However, more complex scenarios such as disease and yield prediction models require detailed soil, land characteristics, and weather information [8]. ML models increase in quality with heterogeneity of datasets.

An intuitive approach toward this problem of valuable and scarce information toward data sharing and reusing is data marketplaces [9]. However, a simple selling platform for datasets and models has limitations as the problem of eroding property claims. However, why are stakeholders of the plant phenotyping ecosystem reluctant to share data or collaboratively acquire, process, share, and utilize data?

A collaborative approach that focuses on data and processing allows the sharing of generated heterogeneous and generally applicable ML models, thus addresses the need described. Therefore, we argue in favor of a collaborative approach for the acquisition and processing of phenotype-related datasets as well as the training of subsequent artificial intelligence (AI) models.

This manuscript describes the limitations of state-of-the-art approaches for data processing and sharing to develop AI-driven applications for plant phenotyping and precision agriculture. It addresses the identified potential of an introduced distributed ledger technology with a smart contract-driven mechanism.

## Limitations to Overcome

Major concerns defined by researchers are (a) non-standardized routines for data acquisition, processing, and cleaning; (b) the risk of eroding property claims; (c) data misuse; and (d) lack of data harmonization and metadata [10]. Some of these concerns, like the lack of standards and metadata, can be faced by implementing the MIAPPE or the ISA-TAB (Investigation/Study/Assay tab-delimited format) standard. Nevertheless, standardization can only overcome some barriers by lowering the hurdle of usability. The need to avoid data getting lost by private copying, misuse, or the missing incentive system is, up to now, not solved. First, the incentive to share data is limited by a lack of accountability (what happens to the data once it is shared with unknown entities, e.g., competitors) and an unclear legal situation with respect to copyright-related aspects. Finally, collaborative sharing of digital information is prone to lead to a network of participants where few contributes to the majority of input. In contrast, most participants only benefit from the network but do not contribute to its well-being, e.g., free-loader [11].

## Toward an Open Data Sharing Ecosystem for Plant Phenotyping Data

In recent times, distributed ledger-based data marketplaces for sharing, distributing, and capitalizing on data gained popularity [12–14]. This is mainly due to their ability to prevent unauthorized access to shared data, accountability in the context of data sharing/access, as well as a variety of incentive structures for data sharing with an emphasis on creating shared, secure, and decentralized ledgers (https://oceanprotocol.com/; https://chain.link/). Initial studies even suggested the suitability of such approaches for the demands of plant phenotyping by proposing a data access control management system for plant phenotyping [15]. A distributed ledger-based infrastructure ensures a proper connection between the data use and reward and the data management decision to avoid eroding property claims.

**Fig. 1.**
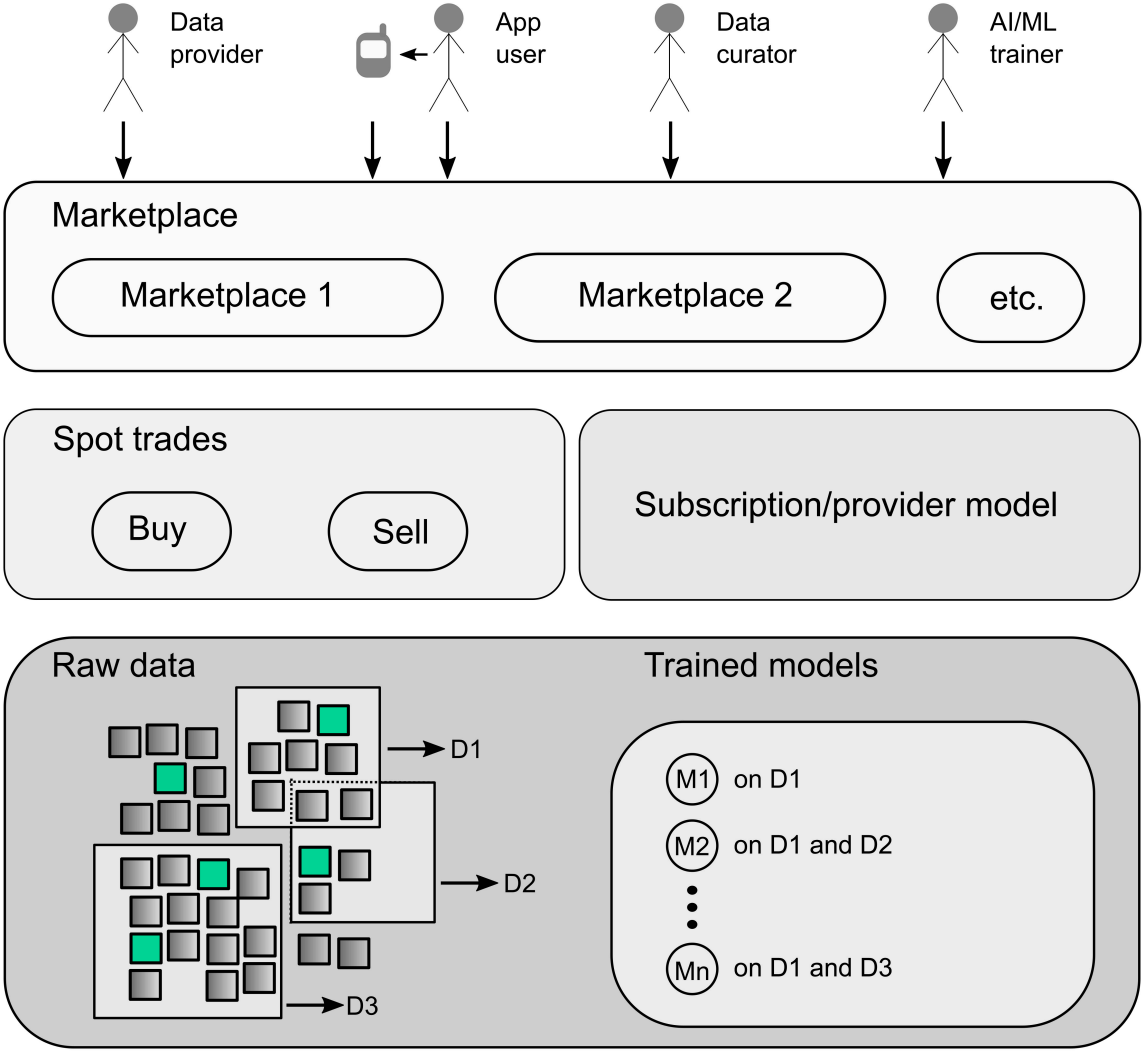
Example of a marketplace ecosystem. Users’ participation covers the data provider, the app/graphical user interface user, the data curator, and the AI/ML trainer. Marketplaces support a buy/sell option and a subscriber/provider option. Available AI/ML models cover different subsets of the training data.

The opportunities of a distributed ledger-based solution go far beyond financial gains. These blockchain-based smart contract-driven ecosystems allow the enactment of collaboration and electronic governance within automated, globally available, heterogeneous sociotechnical e-governance systems with loosely coupled, peer-to-peer-resembling network structures and are characterized by their dynamic, continuously changing, interoperable, open, and distributed nature [16]. Thus, we propose that an open, collaborative ecosystem for plant phenotyping and precision agriculture datasets can be designed as illustrated in Fig. 1. The stakeholders of the ecosystem are data users, data providers, data curators, and creators of AI models. Data providers acquire data and send it into the existing data pools. Note that a user can also act as a data provider by using an application that uses an AI/ML model, while new data are added to the data pool. Data curators process incoming data, remove low-quality input, and add metadata as well as labels when applicable. Data curators also review other curators’ data for quality assurance. Finally, AI/ML trainers access and utilize ecosystem datasets to develop, train, test, and evaluate AI/ML models, which are subsequently provided to the ecosystem.

Users may invoke one-time-only enactments (buy/sell) of data or choose a subscription model to gain access to a continuously updated and curated dataset and an AI/ML model. Users either directly interact with a marketplace or via an external user interface that provides an AI/ML model prediction trained by using the underlying datasets. In the agricultural context, this could be the detection of a disease or a forecast of yield, considering adequate training sets from the geographical position.

Instead of just a single marketplace that may lead to monopolies/oligopolies (e.g., Android/iOS marketplaces) [17], we propose a set of interoperable, open, and distributed marketplaces where stakeholders can choose any of the marketplaces but still gain access to the whole ecosystem. Data and models are both bought and sold by the stakeholders as part of a spot trade or gain continuous access via a subscription model. Various market mechanisms may apply to provide discounts to those who contribute to the well-being of the ecosystem by providing and curating datasets or new AI/ML models.

A one-stop platform for value exchange, collaborations, and business enactments is preferable over a fragmented, dysfunctional ecosystem of marketplaces “with deliberately forced, or functional lock-ins that lead to the formation of self-contained data and service silos such as Tesla, Google, or Amazon” [16]. Moreover, an interoperable smart contract-driven ecosystem may be operated as a joint venture of various stakeholders, which includes built-in e-governance mechanisms, thereby constituting a neutral territory for all stakeholders while also reducing dependency on intermediaries [18].

Despite serving as the foundation of our proposal, a smart contract-driven ecosystem also poses challenges. First, the described approach constitutes a multistakeholder setup that not only may consist of publicly available transactions, smart contracts, and data but also may rely on privacy-sensitive information, which raises privacy concerns [19]. Therefore, a complementary setup of public and permissioned blockchains is required, which also requires appropriate tools to create, deploy, and manage complex orchestrations of smart contracts. Second, smart contracts allow for value transfer, e.g., payments, via tokens. However, most real-world business transactions rely on fiat currencies. Thus, a settlement mechanism to handle token and fiat payments is necessary. Third, smart contract-based transactions and collaborations are still subject to legal compliance [20]. Fourth, integrating smart contracts into a legacy system is challenging and requires special care.

## Potential Advantages for Modern Agriculture

**Fig. 2.**
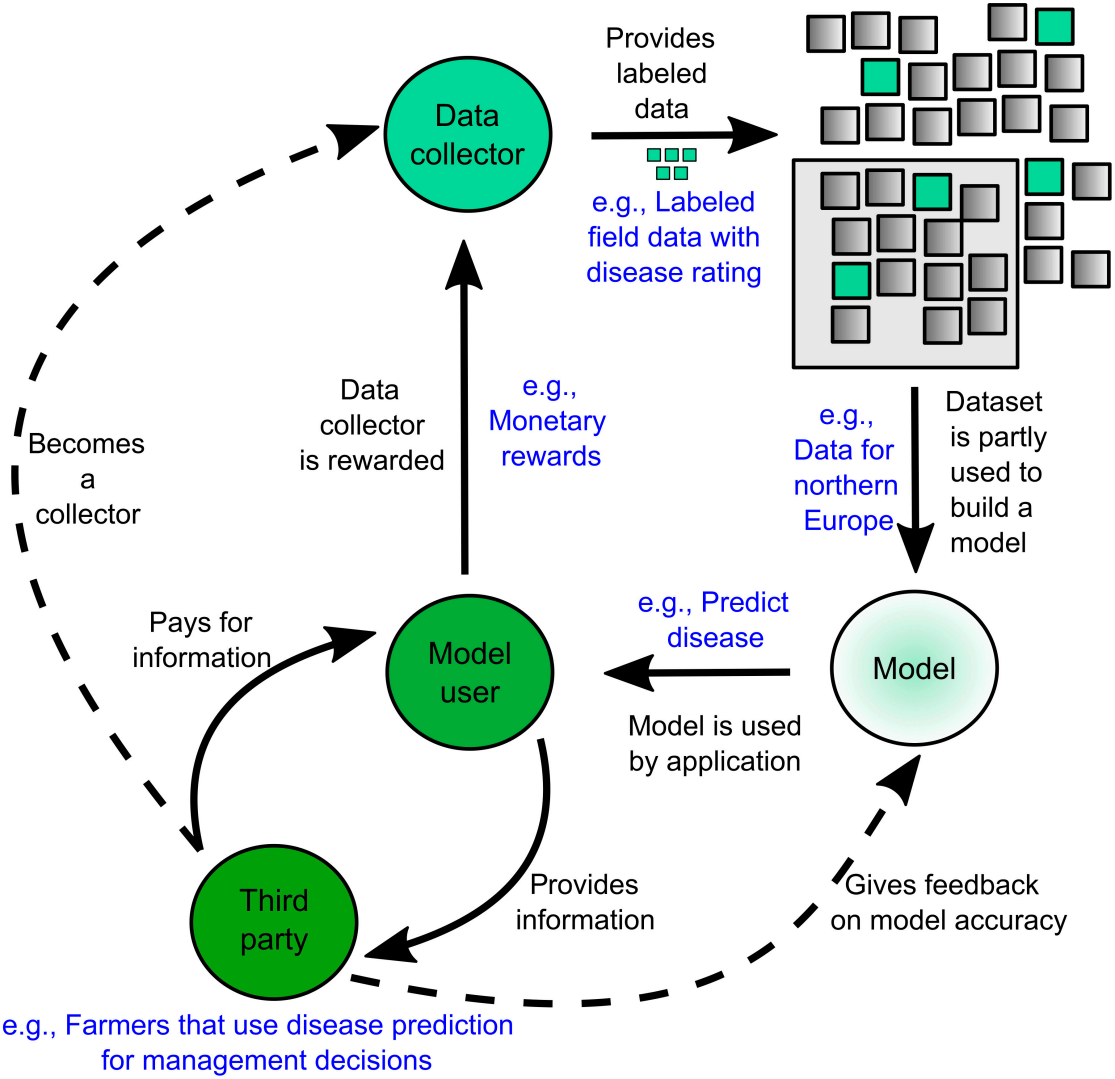
A use case for the data marketplace ecosystem in modern agriculture. Data can be used in parts to train a ML model, which are provided by a model user to a farmer. The example is visualized by the use case of disease prediction based on remote-sensing field data.

Figure 2 embeds the proposed approach into an agricultural use case, disease prediction for crops on a practical field site. Here, field data from unmanned aerial vehicle flights or satellite images are used to train a model to predict the estimated crop disease severity/incidence on the field. The approach can be generalized to any other plant-based use cases such as yield prediction, behavior prediction, or nutrient modeling, which utilize ML models.

Four roles were defined: the data collector; the ML model; the model user, e.g., the Application Programming Interface of a mobile phone application; and the farmer. The data collector prepares the datasets using standards, meta information, and data harmonization and sends them to the data storage. Whenever the ML model is used, completely or even partly, the data collector is rewarded by the model user. The model user provides information to the farmer who pays for it. Farmers can also become data collectors by providing reference data such as disease reference data (position, incidence, and severity). Furthermore, they give feedback about the accuracy/quality of the model.

Following this, different effects on agriculture, as it is seen today, can be defined: (a) a possible new role for a farmer as a supplier for ML training data. By providing more and better training data, the quality of forecast and prediction tools can be improved, which leads to (b) the possibility to increase the reliability of agricultural forecast systems, which enables the adaption of management routines and crop rotation decisions when, e.g., implementing disease forecasts. Aspect (b) is also true for nondistributed approaches, as they are based on exact prediction systems. Nevertheless, a distributed ledger-based approach will tremendously increase the accuracy of ML-based predictors and can help push these aspects into daily use.

## Conclusion

This manuscript describes an approach that enables scientists and plant-phenotyping entities to improve the availability of scientific data by introducing a distributed ledger-based tracking of data that is integrated into a broader ecosystem that provides different incentives to its stakeholders. It depicts one possible solution to overcome various island solutions for data storing and sharing by connecting edited datasets with metadata from different actors. It helps to overcome the current bottleneck of ML models that can tremendously increase their accuracy by using vast amounts of labeled training data. Therefore, the roles of the data collector, modeler, and model end-user are defined. The workflow based on a distributed ledger approach is explained using a practical example of an agricultural disease prognosis system.
